# Melkersson-Rosenthal syndrome in a patient with tubercular panuveitis

**DOI:** 10.4103/0301-4738.58481

**Published:** 2010

**Authors:** Kalpana Babu, Prashanta V Gundannavar, Vidya Satish, Venkatesh C Prabhakaran

**Affiliations:** Vittala International Institute of Ophthalmology & Prabha Eye clinic and Research center, Bangalore, India

**Keywords:** Isolated eyelid edema, Melkersson-Rosenthal syndrome, tubercular panuveitis

## Abstract

We report a rare presentation of Melkersson-Rosenthal syndrome in a patient with tubercular panuveitis. A 45-year-old male being treated with antitubercular therapy for tubercular panuveitis presented with unilateral, non-pitting right upper eyelid edema. Excision biopsy showed granulomatous inflammation involving the lymphatics. Immunohistochemistry confirmed the presence of histiocytes around the lymphatics.

Melkersson-Rosenthal syndrome (MRS) is characterized by recurrent, relapsing orofacial edema, facial palsy and a fissured tongue.[1,2] It is a rare, non caseating granulomatous disease of unknown cause. It was described by Melkersson as a syndrome of recurrent facial paralysis and edema. Rosenthal added the third feature, furrowing of the tongue. The furrowed tongue is the least common finding. The complete triad is very rare and monosymptomatic or sequential involvement is more common.[1,2] Histopathology showing the presence of perilymphatic granuloma, lymphangiectasia and granulomatous lymphangitis is diagnostic of the above syndrome.[3] Occasional reports of isolated painless, non-pitting and unilateral eyelid edema due to MRS has been described.[1,2,4] We report a case of isolated eyelid edema due to MRS, confirmed histopathologically, in a patient with tubercular panuveitis.

## Case Report

**Figure 1:**
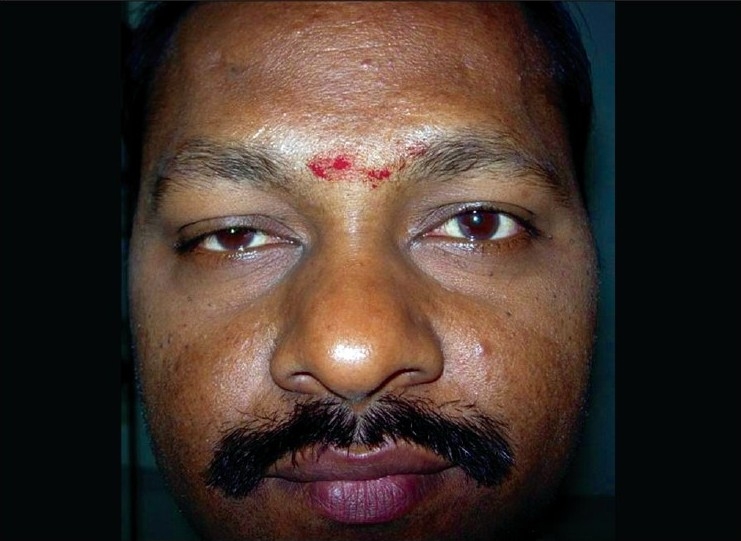
Photograph of the face showing right upper eyelid edema

**Figure 2:**
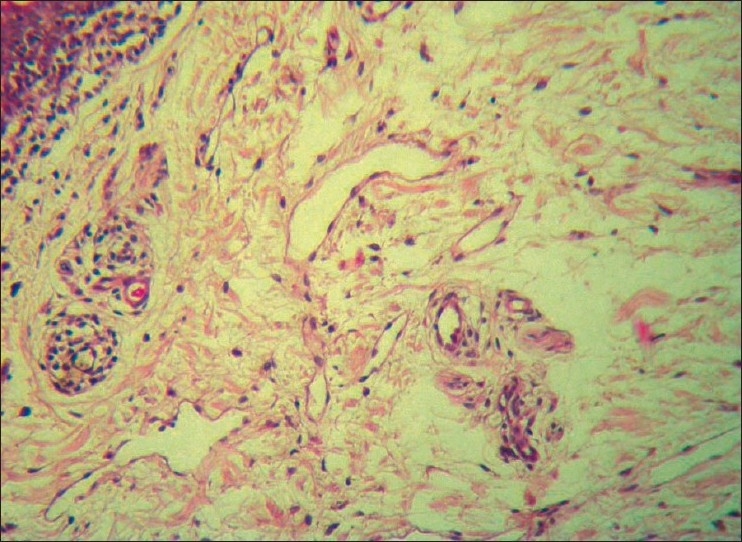
Photomicrograph showing the thin-walled lymphatic vessels with lymphocytes infiltration (Hematoxylin and Eosin, ×20)

**Figure 3:**
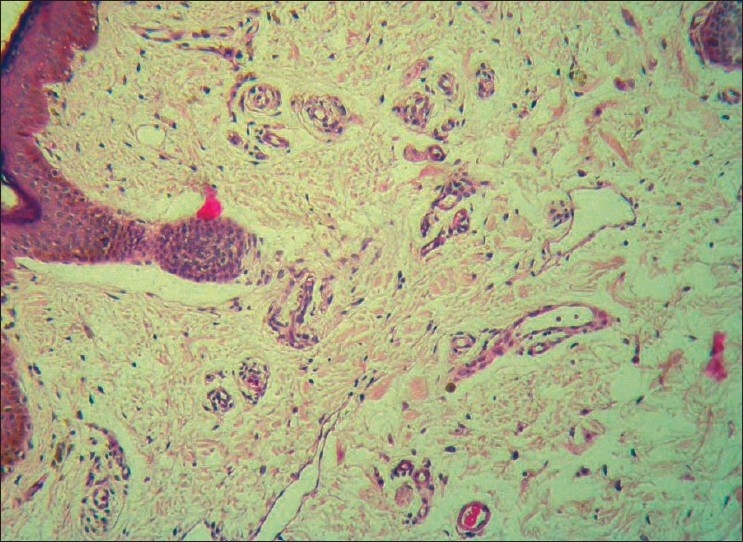
Photomicrograph showing intralymphatic histiocytic infiltration (Hematoxylin and Eosin, ×20)

**Figure 4:**
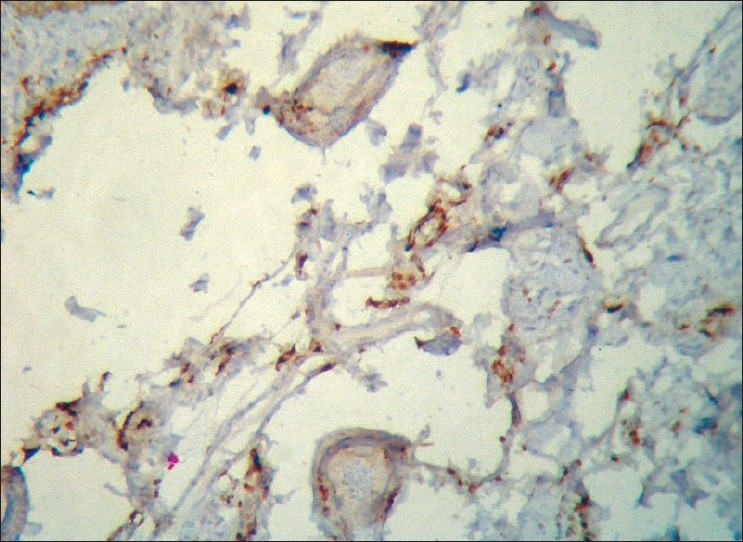
Photomicrograph showing positivity to CD68 marker confirming the presence of histiocytes (Immunohistochemistry, ×20)

**Figure 5:**
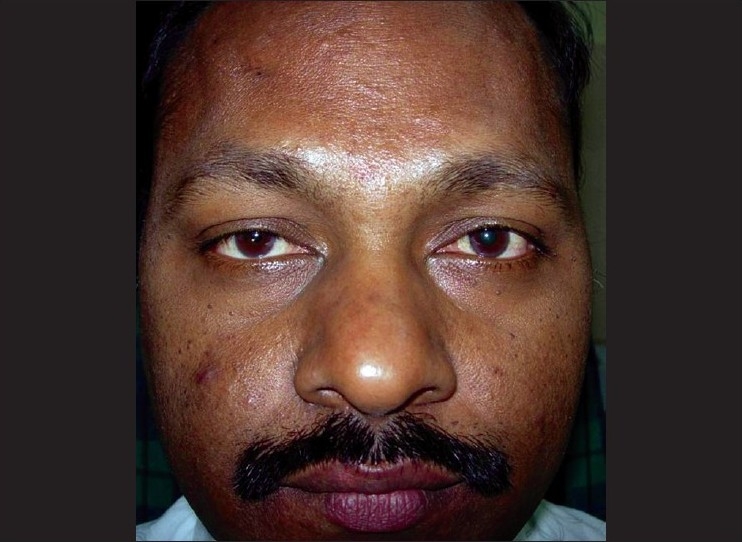
Postoperative photograph of the face showing improved cosmesis

A 45-year-old male was being treated for tubercular panuveitis in both eyes since one year with four-drug regimen of antitubercular therapy (ATT) which included isoniazid, rifampicin, pyrazinamide, ethambutol for two months followed by isoniazid, rifampicin and pyrazinamide for ten months. ATT was given for a possible tubercular etiology on the basis of a strongly positive Mantoux test (25-mm induration), a calcified nodule on chest X-ray and demonstration of *Mycobacterium tuberculosis (MTb)* genome by polymerase chain reaction (PCR) on the vitreous sample. He had no recurrences and there was complete resolution of ocular inflammation following treatment with ATT. During his follow-up visits, he gave a history of intermittent right upper eyelid swelling since the last four months, which was more prominent during the day. Past history revealed a history of recurrent left-sided facial edema five years previously for which no investigations were done at that point of time. He did not have any facial nerve palsy or furrowed tongue at presentation. On ocular examination, a firm, non-tender and non-pitting edema involving the entire right upper eyelid was seen. [Fig. 1] His best corrected visual acuity was 20/20 in both eyes. Slit-lamp examination was unremarkable. Fundus examination showed areas of healed retinal vasculitis and multifocal choroiditis. Thyroid function tests, renal function tests and computed tomography of the cranium and orbits were normal. A right upper eyelid biopsy was done. Histopathology showed many empty, ectatic, thin-walled vessels consistent with lymphatics [Fig. 2]. An inflammatory infiltrate consisting of lymphocytes and histiocytes was seen surrounding these vessels. One area of section showed intralymphatic histiocytic infiltration [Fig. 3]. Immunohistochemistry was positive for CD68 marker confirming the presence of histiocytes [Fig. 4]. Quantitative PCR (qPCR) on the paraffin-embedded skin biopsy specimen was negative for *MTb*. The excision of a part of eyelid skin improved the patient's appearance and the patient preferred to be under observation [Fig. 5].

## Discussion

In our case, the complete clinical triad of MRS was not present. Although eyelid edema due to MRS is an unusual entity, the clinical findings may be difficult to differentiate from blepharochalasis or thyroid-associated ophthalmopathy. There is an isolated report of a possible association with tuberculosis.[3] However, in our case, the paraffin-embedded skin biopsy specimen did not demonstrate the *MTb* antigen by qPCR. The association in our case with tubercular panuveitis may be casual. Treatment is challenging and includes intralesional usage of corticosteroids and or oral treatment with dapsone, clofazimine, tetracycline, clindamycin and methotrexate without any reproducible improvement. A combination of surgical debulking and intralesional steroid therapy has been advocated to improve cosmesis. Because of its rarity, this syndrome is usually ignored and misdiagnosed. MRS may be considered in all cases of isolated eyelid edema and a diagnostic incision biopsy may be performed to establish a diagnosis.
